# Supplementary material for: Degenerating intervertebral discs in the streptozotocin-high-fat diet model of type 2 diabetes show extensive inflammation
Source: Dis Model Mech. 2025 Nov 26;18(11):dmm052384. doi: 10.1242/dmm.052384 (PMC12690530; doi:10.1242/dmm.052384)
Supplement: Supplementary information [file dmm-18-052384-s1.pdf]

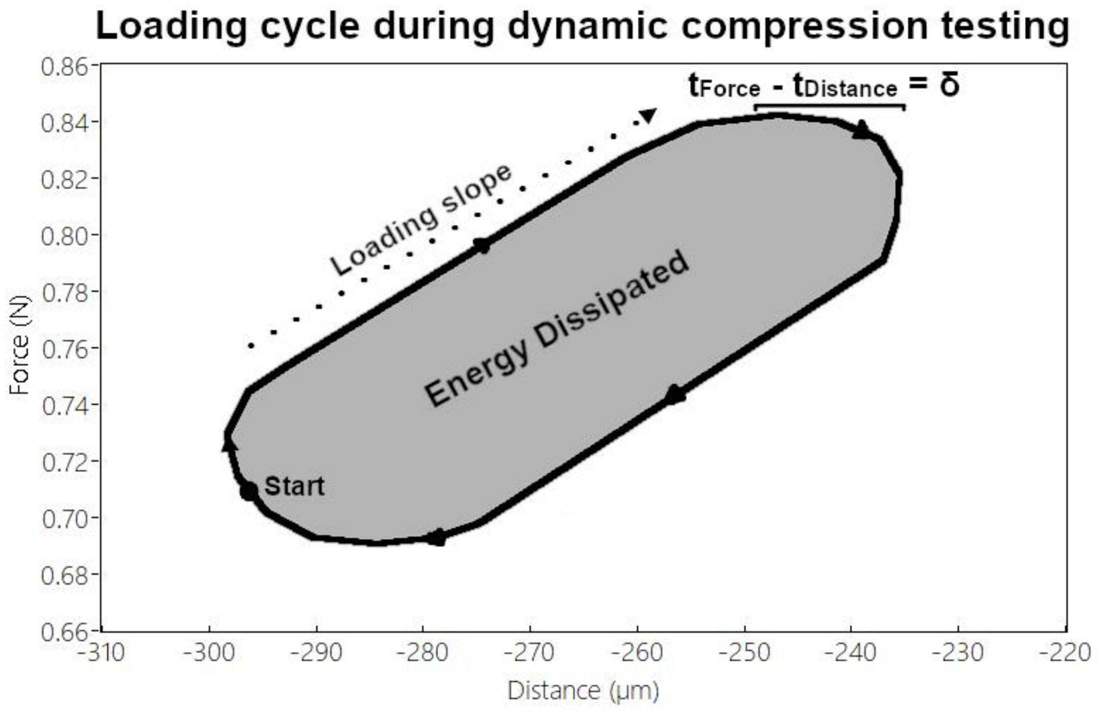

Fig. S1. Representative force-displacement curve of the dynamic loading regimen used in mechanical testing. The force-displacement curve here illustrates the loading hysteresis in these samples, consistent with behavior of a viscoelastic material.

Disease Models & Mechanisms • Supplementary information

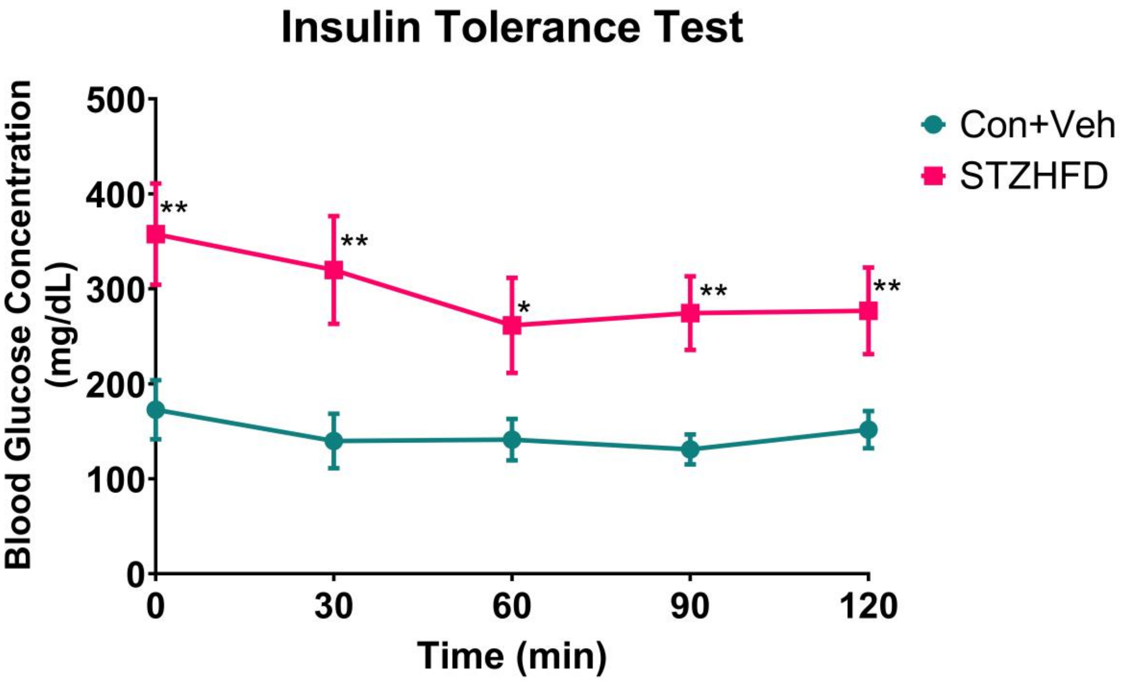

Source of Variation	% of total variation	P value
Time x Diabetes	2.423	0.0672
Time	7.352	**0.0057**
Diabetes	75.24	**0.0004**
Subject	9.228	**0.0004**

Two-way ANOVA
$n_{Con+Veh}$ = 4
n_{STZHFD} = 4

Fig. S2. Insulin tolerance test of STZ-HFD mice shows insulin resistance, not insulin dependence. Despite receiving 2U/kg I.P. insulin (Humalin R), STZ-HFD mice do not respond with a reduction in blood glucose concentration. Consequently, STZ-HFD mice have elevated blood glucose when compared to non-diabetic controls at all timepoints during the insulin tolerance test.

Disease Models & Mechanisms • Supplementary information
